# Supplementary material for: Rapid turnover of life-cycle-related genes in the brown algae
Source: Genome Biol. 2019 Feb 14;20:35. doi: 10.1186/s13059-019-1630-6 (PMC6374913; doi:10.1186/s13059-019-1630-6)
Supplement: Supplementary file 3 — Protein alignments, corresponding Gblocks html file and CDS sequences for the lineage conserved gametophyte-biased genes. (ZIP 2109 kb) [file 13059_2019_1630_MOESM3_ESM.zip › Con_GA_alignments/Ec-14_005390.aa_ali.fasta-gb.htm]

Ec-14\_005390.aa.fasta.trans.aa\_ali.fasta


## Gblocks 0.91b Results

Processed file: **Ec-14\_005390.aa.fasta.trans.aa\_ali.fasta**  
Number of sequences: **2**  
Alignment assumed to be: **Protein**  
New number of positions: **1190** (selected positions are underlined in blue)

```
                         10        20        30        40        50        60
                 =========+=========+=========+=========+=========+=========+
Ec-14_005390     ----------------------------------------------------MTRAAAVN
MSTRG.39725.1    DRPSFTFDMVATAEPRHRGVGATKAVPPTLKSVRPKLFRRASLTSWGKNNLTMTRAAAVN
                                                                     ########


                         70        80        90       100       110       120
                 =========+=========+=========+=========+=========+=========+
Ec-14_005390     VKELMLTVQQQKYEDRDKAAQREISMKESDEGQQSIRRRDERRSRWLLRSTVRREFLQNW
MSTRG.39725.1    LKELMLTVQQQKYEDRDKAAQRQISQKESCEGEQSILKRDERRSRWLLRSSAKREFLQNW
                 ############################################################


                        130       140       150       160       170       180
                 =========+=========+=========+=========+=========+=========+
Ec-14_005390     EREGRAKWNSNQASLCRYERRLLNYELAVKATTAARKRISRNRHSNDEADGIRWFEHNLD
MSTRG.39725.1    ERDGRRNWNANQAALCRYERRLLNYELAVKATAQARKRLSRARHFNDETEGIQWFEHNLD
                 ############################################################


                        190       200       210       220       230       240
                 =========+=========+=========+=========+=========+=========+
Ec-14_005390     RLGLDPGGGSVDGGGGAGA----LSPDKETPASFRSRLVRAVLDQNFVPSSNAEAMAELR
MSTRG.39725.1    RLGLDPGGGGAGGGPGGGAAGSGVAPDKETSATFRSRLVRTVLDQKFVPSSNAETMAELR
                 ###################      ###################################


                        250       260       270       280       290       300
                 =========+=========+=========+=========+=========+=========+
Ec-14_005390     FRGVTGRRGRKEREDRRVKTEVDQRRATAETDAKRRAEKHLKEMLDKGRERQEAAAACWG
MSTRG.39725.1    FRGTTGRRGRQEREHRRVKTDVDQRRAQAETDAQRKAENDLKGMLNEGRERREAAAASWR
                 ############################################################


                        310       320       330       340       350       360
                 =========+=========+=========+=========+=========+=========+
Ec-14_005390     ERRNLERKAIADKRRFAEMAAAQEDAFETAFNARADCTRQSYEERSAERMANNEALRIRV
MSTRG.39725.1    AEHERQQKALSDEKRFAEKSAALDGAFEVAFNARASHVRQSYEESKDAREAKEDALRVEL
                 ############################################################


                        370       380       390       400       410       420
                 =========+=========+=========+=========+=========+=========+
Ec-14_005390     QQTRTEKRRRAETMCAEIVHKMSDLAVVSSELRASRGGVPLPPTAWAHLKRRFCSPEPFF
MSTRG.39725.1    HETNEKKRRGIETMCRHVAHKLSDLAVVASEARVLQGGIPLAPTTWANLKRWFCSPEPFF
                 ############################################################


                        430       440       450       460       470       480
                 =========+=========+=========+=========+=========+=========+
Ec-14_005390     VDSTPPEPTPEPRDPVLDAKAFLESQNLDRCEGLWRLREDVPAELFGAPSPLAKALGVAR
MSTRG.39725.1    VDATPPEPLPEPSDPVLDAKVAVESLNLDRHEGPWRLRWDPQQIPPIGPSPLSRALAVAR
                 ############################################################


                        490       500       510       520       530       540
                 =========+=========+=========+=========+=========+=========+
Ec-14_005390     GLVEASGSTPREAPNYGRNSRSGDDKATRPDVRLVLLSRRDGLFDLAAELGQWVGVYVCS
MSTRG.39725.1    ELSEASGSGPREAP----TCTLGGGE---MSVRLVLLGRRDDMDNLLEGLGRWTRLYVCS
                 ##############                 #############################


                        550       560       570       580       590       600
                 =========+=========+=========+=========+=========+=========+
Ec-14_005390     METALECAMEVGAEAAASDDKGGKSRKGSISGRKSSVGGENSGENAPGVDAETAAEEAEK
MSTRG.39725.1    LETALECAMAVGAEIAASEGKGTKGRKASALG-----------KNVVGSDAEPVDTESEE
                 ################################            ################


                        610       620       630       640       650       660
                 =========+=========+=========+=========+=========+=========+
Ec-14_005390     ATARRCFHPDATDEDTAAFKAAAMAYHALRTHPKKASAPVPLATTTDLLVKHLSCRAPSG
MSTRG.39725.1    MTIQRCFHPEATEETVAAFKEAAVAYHALRTNPKKASTPVPSASTTDLLVKHLECRAPRG
                 ############################################################


                        670       680       690       700       710       720
                 =========+=========+=========+=========+=========+=========+
Ec-14_005390     RGWILVGYPTTLLESKLLENALSSYTDDEVAAELGTAGKASKSDAKTKKMSLALQQQEEP
MSTRG.39725.1    RGWILVGYPKSLLESKMLENALSGYADEEVATELGTSGKASKADSKTKKGSIVPQQQEDR
                 ############################################################


                        730       740       750       760       770       780
                 =========+=========+=========+=========+=========+=========+
Ec-14_005390     PRALPRSGLDAVLSLAEPCLSSPRVGALPQDSVAGIQGDAEANAPNEDLAAASTTDEADG
MSTRG.39725.1    SQTPLRSGLDAILMLTSLRSTAPRRTSHPSDLRTTSEDGGAATVSSDELADAPSTQEAGA
                 ###############################          #################  


                        790       800       810       820       830       840
                 =========+=========+=========+=========+=========+=========+
Ec-14_005390     NGGGQDEEDVERSKCLAERRAQIAWLQGFEGGHLSCDVPNEANNERLLETLFLLVNIAQN
MSTRG.39725.1    IHQSKLDEDVVDDSSPAQKDAQSRWWQGFEGGHLACDVPDEANDERLLETLFLLVNAAQI
                        #####################################################


                        850       860       870       880       890       900
                 =========+=========+=========+=========+=========+=========+
Ec-14_005390     RKDEHEFWQKEPALSRRNSEVEATQALEGGGSSEPPSDTKSHSTIAMVTDRPSPLAVPLS
MSTRG.39725.1    RKDEHQVFEEA---ARKSQEERAAQALESGSAAEPDGGANSPSTTATAIDRPSPLTIPLT
                 #####          #############################################


                        910       920       930       940       950       960
                 =========+=========+=========+=========+=========+=========+
Ec-14_005390     ASVERLRRERRGNMSQSTRIQLEALMGDRELDLEGVRSAVSQAS-------------VLA
MSTRG.39725.1    SCLEDSRRQRRRRMSHATRIWVEALTGDRELDLDGIRTVISHANARKEGPGASAVTITLG
                 ###########################################               ##


                        970       980       990      1000      1010      1020
                 =========+=========+=========+=========+=========+=========+
Ec-14_005390     ADTTEGACSP--QQTALRKLEAESPAPSVIDWASTAEFSFSKELRNPDDRWATACLRLEA
MSTRG.39725.1    STTSPPSASSPCSTQALRKLEAECSALFVVDWAAAAEFSFSTELRNPDQRWTAACNKLEG
                 #########      #############################################


                       1030      1040      1050      1060      1070      1080
                 =========+=========+=========+=========+=========+=========+
Ec-14_005390     QMAGWLARRRDALEAMQSRRSRGDRATRDSRDD-QSITASVPPAEGDCVWEALEAFQLEA
MSTRG.39725.1    EIASWLERRRGTLPAGRSTRSRGGPAPWDGRGCPSAATAAVAMQKENCVWQALEAFQLEA
                 ###############################      #######################


                       1090      1100      1110      1120      1130      1140
                 =========+=========+=========+=========+=========+=========+
Ec-14_005390     GDVVDDRHSSAETIVGQIDGEADRKVRAWIGDVASAATDLCAAERATYLETVDTLCVFDR
MSTRG.39725.1    GDVVDDRHSSVEAVMKEIGEEAGRRVRAWTRNVENAAAGLCVVERATHLETVAALGVLDR
                 ############################################################


                       1150      1160      1170      1180      1190      1200
                 =========+=========+=========+=========+=========+=========+
Ec-14_005390     LFGLEEDPNDNTSSAQTIREATYAAADALVEEVTKAR--RRFAAVGSCLEGEVSRALEQV
MSTRG.39725.1    LFGLEDD--ERAGTADQSKGAACAAVDALGKELSTALGDRLYPSEGGAFKEELRRTIEEV
                 #######       ######################   #####################


                       1210      1220      1230      1240      1250      1260
                 =========+=========+=========+=========+=========+=========+
Ec-14_005390     YQRIGTTSPAHGPSQEPVTLLNAGCETRQENPLPQAIEGVEE---FSSSAGQSSDWTATN
MSTRG.39725.1    YEECRATSPAHDPSQKLAALQALDSASQPEAPSSQRCQDVGDGLVHGDDIDEASICRVSS
                 ####################         ###########             #######


                       1270      1280      1290      1300      1310      1320
                 =========+=========+=========+=========+=========+=========+
Ec-14_005390     DTPTKRKAETSVTIEECGESRASEALKAAIWRCRQTYICRLRGVVMRLLRAVGDCEAKVL
MSTRG.39725.1    ATISTTTAKFASGSSKGDTEEEAGALAIAVWRCRHTYICRLRGLVMRMLRAIEHCEAKVS
                 ########                ####################################


                       1330      1340      1350      1360      1370      1380
                 =========+=========+=========+=========+=========+=========+
Ec-14_005390     SMRLALRRLKRRRVQLEHEGISAATATVRRALQECDHVILADMLENGIPLWIEDDETFAA
MSTRG.39725.1    SMLAALRRLKRHRVQVEHEGISTGTAVVRRALEECDFTAIADILENGIPVWVEDDETFAP
                 ############################################################


                       1390      1400      1410      1420      1430      1440
                 =========+=========+=========+=========+=========+=========+
Ec-14_005390     DPFVALPWGMNGLGLPCIASLADSLQRSLSGAEGVPGSNTVMAVADLSRAVRAWLHSKIT
MSTRG.39725.1    D-----------------------------------------------------------
                 #                                                           


                       1450      1460      1470      1480      1490      1500
                 =========+=========+=========+=========+=========+=========+
Ec-14_005390     ERNREGIMSDIPRAWISSGNGYIDTLCGATADGMDDVSWRRLIHCLLLGHVRSLPTLDDL
MSTRG.39725.1    ------------------------------------------------------------
                                                                             


                       1510      1520      1530      1540      1550      1560
                 =========+=========+=========+=========+=========+=========+
Ec-14_005390     ISLRNALCLKASTVSQTSVGIGPAADAASPDVRSSTTQMPTLRIAQRHFVGTPLWFEQQR
MSTRG.39725.1    ------------------------------------------------------------
                                                                             


                       1570      1580      1590      1600      1610      1620
                 =========+=========+=========+=========+=========+=========+
Ec-14_005390     DEVKHGEERADRDASQLIRKVYSQVWGGDDGRVDGMDLLLTLCAVPNAGAGGDVSLGEDE
MSTRG.39725.1    ------------------------------------------------------------
                                                                             


                       1630      1640      1650      1660      1670      1680
                 =========+=========+=========+=========+=========+=========+
Ec-14_005390     EGVWDTGIPFSAGLFRAFFMLAETSSSGPHNDTIVANSNREVASAGTVASRVAAPSSSAG
MSTRG.39725.1    ------------------------------------------------------------
                                                                             


                       1690      1700      1710      1720      1730      1740
                 =========+=========+=========+=========+=========+=========+
Ec-14_005390     NMQDVAAGATGIMSSFRRDGVFPRVSLPHLRAILGHGSRMTPADVSSVCELVAKREQARR
MSTRG.39725.1    ------------------------------------------------------------
                                                                             


                       1750      1760      1770      1780      1790      1800
                 =========+=========+=========+=========+=========+=========+
Ec-14_005390     HQDEKQGHGVKQDTAAEVVVGTEEATGAENEPSVARQIMGTGAETAGATEEDCDVAPDTT
MSTRG.39725.1    ------------------------------------------------------------
                                                                             


                       1810      1820      1830      1840      1850      1860
                 =========+=========+=========+=========+=========+=========+
Ec-14_005390     ATGDPGGGRGIAVRQKEPPLTVSFDVVSECEVVRDWVRKGAYTLPTFGVLSGRGQGTVNM
MSTRG.39725.1    ------------------------------------------------------------
                                                                             


                       1870
                 =========+======
Ec-14_005390     LGKTSHSFGESIGESQ
MSTRG.39725.1    ----------------
```

```
Parameters used
Minimum Number Of Sequences For A Conserved Position: 2
Minimum Number Of Sequences For A Flanking Position: 2
Maximum Number Of Contiguous Nonconserved Positions: 8
Minimum Length Of A Block: 10
Allowed Gap Positions: None
Use Similarity Matrices: Yes
```

```
Flank positions of the 15 selected block(s)
Flanks: [53  199]  [206  494]  [512  572]  [585  751]  [762  778]  [788  845]  [856  943]  [959  969]  [976  1051]  [1058  1147]  [1155  1176]  [1180  1220]  [1230  1240]  [1254  1268]  [1285  1381]  

New number of positions in Ec-14_005390.aa.fasta.trans.aa_ali.fasta-gb:  1190  (63% of the original 1876 positions)
```
